# Supplementary material for: Building de novo reference genome assemblies of complex eukaryotic microorganisms from single nuclei
Source: Sci Rep. 2020 Jan 28;10:1303. doi: 10.1038/s41598-020-58025-3 (PMC6987183; doi:10.1038/s41598-020-58025-3)
Supplement: Supplementary file 1 — Supplementary information. [file 41598_2020_58025_MOESM1_ESM.pdf]

Building *de novo* reference genome assemblies of complex eukaryotic microorganisms from single nuclei

Merce Montoliu-Nerin, Marisol Sánchez-García, Claudia Bergin, Manfred Grabherr, Barbara Ellis, Verena Esther Kutschera, Marcin Kierczak, Hanna Johannesson, Anna Rosling

Supplementary information, 9 figures and 6 tables

Figure S1. Spores of *C. claroideum/C. luteum* (SA101)
Figure S2. Scatter plots from the FACS sorting during method development
Figure S3. Agarose gel of PCR products of particles in R1, R2-R4, R4
Figure S4. Scatter plot of particles from one single crushed spore
Figure S5. PCR scoring for fungi and bacteria in amplified single nuclei
Figure S6. Summary statistics for assembled of 1-24 nuclei in assembly workflows 1
Figure S7. Original gel image corresponding to figure S3
Figure S8. Original gel image corresponding to the upper part of figure S5
Figure S9. Original gel image corresponding to the lower part of figure S5

Table S1. Number of particles collected
Table S2. 96 well plate layout for sorting.
Table S3. Summary of PCR scoring for fungi and bacteria
Table S4. DNA concentrations of 24 sorted and amplified nuclei
Table S5. Single nuclei assembly statistics for the assembly workflow 1 and 2
Table S6. Presence of the single copy genes EF1 and RPB1 in the three assemblies

Developing the protocol of single nuclei extraction

Broken spores were observed in a microscope equipped with a fluorescent filter to assess amount, release efficiency and size of the nuclei. Spores were placed on a slide and the excess water was removed with a pipette. A small drop of Gold Slow Fade + DAPI was added and the glass cover was placed pressing gently with the back of a pencil, to crush open the spore without destroying it. Crushed spores were visualized under fluorescent microscope. (Figure S1, Original pictures in OSF Repository[44]).

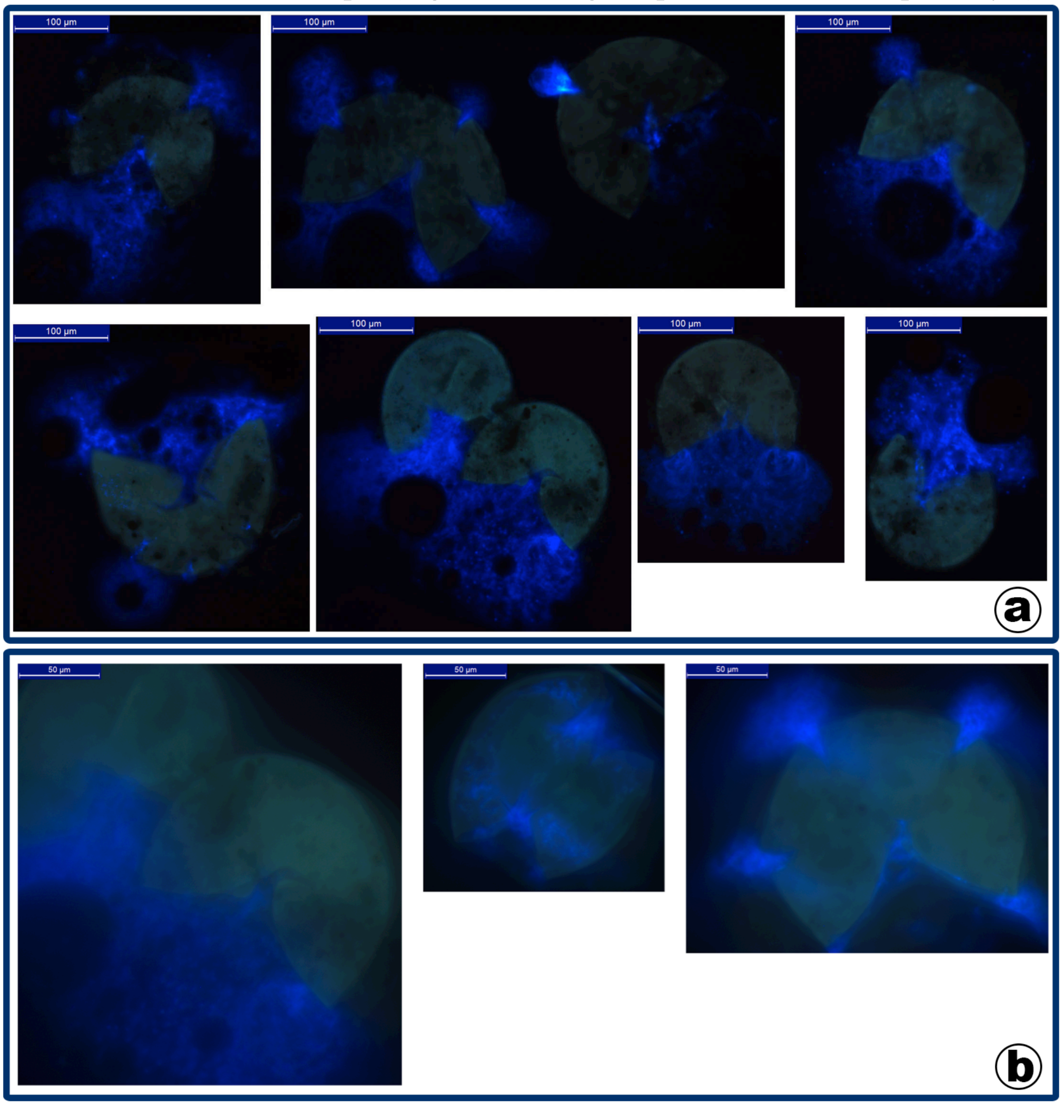

Figure S1. Spores of *C. claroideum/C. luteum* (SA101) at 20X (a) and 40X (b) seen in the microscope after staining with DAPI in order to visualize the nuclei and assess their size.

For the sorting of the nuclei, two tubes with 15 and 25 spores of the isolate respectively, previously extracted and cleaned were crushed in 200 µl of ddH2O, and 200 µl of 1X PBS was added together with 1.25 µl of 200X SYBR Green. After 37 min staining, 100 µl of 1X PBS was added to increase the volume before running the sample in the FACS. FSC on the 488 nm laser was used as trigger, and a 530±20 nm (530/40) band pass filter for fluorescence signal detection. The best resolution of distinctive populations of particles in the scatter plot was obtained when plotting level of fluorescence against the SSC representing granularity (Figure S2).

Three regions were defined in the scatter plot: R1 (a distinctive line with strong fluorescence), R4 (a distinctive community lower fluorescence and lower SSC values than R1), and R2 minus R4 (an undifferentiated area between R1 and the background fluorescence) (Figure S2). Particles appearing in those areas were sorted simultaneously into three different tubes (Table S1). Additionally, two tubes were filled with particles from all the areas in each sorting round, to be used as a positive control.

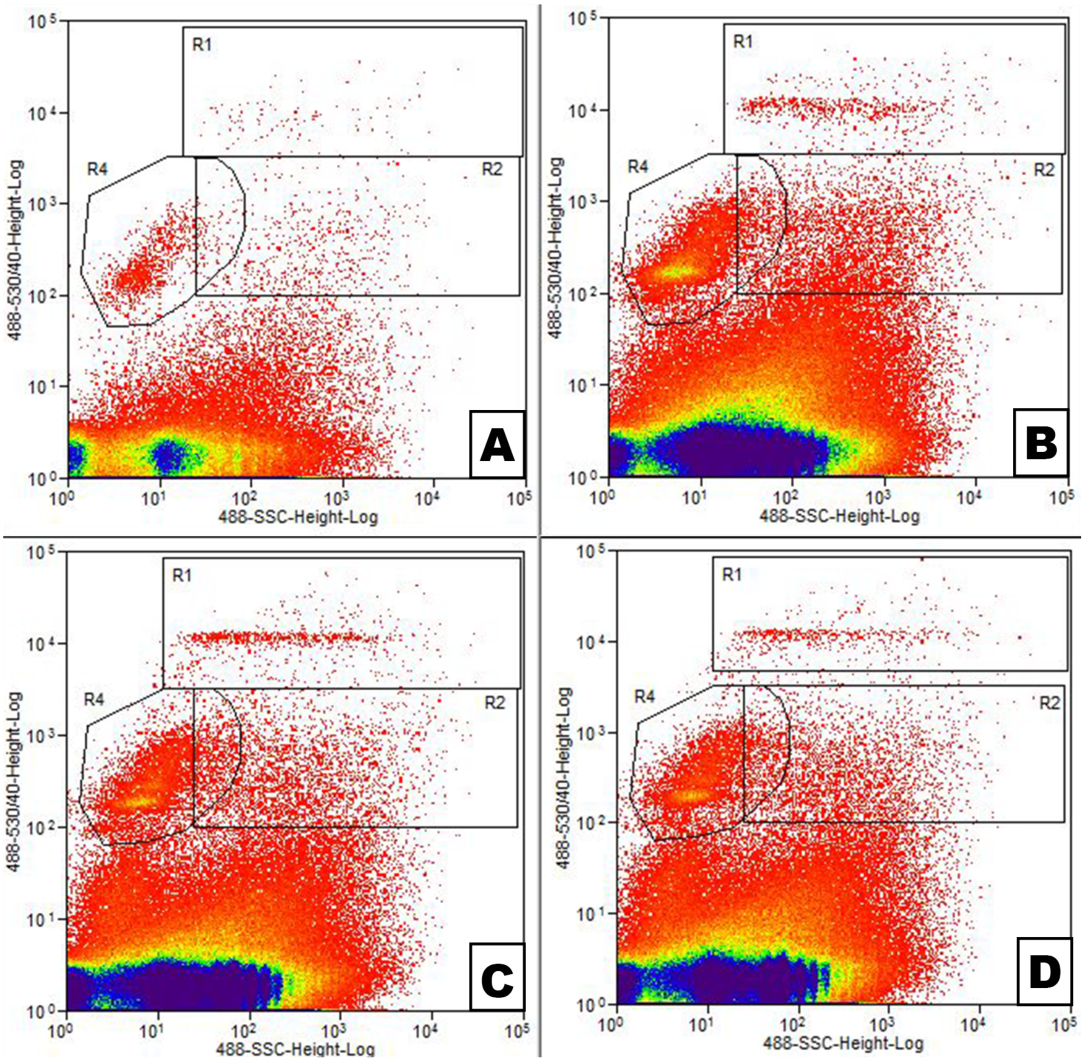

Figure S2. Scatter plots from the FACS sorting of particles from 15 (a, b) or 25 (c, d) crushed spores using Forward Scatter (FSC) as trigger. On the x axis, SSC, on the y axis, the SYBR Green fluorescence. The low-fluorescence part of the scatter plots represents background fluorescence in the spore content, that is either not stained or very lightly stained. a) Scatter plot of from the first quick run identifying the areas to sort from. b) Scatter plot of actual sorting into pools (R1, R2, R4) of particles from the 15 spores (Table S1). c) Scatter plot of sorting into pools (R1, R2, R4) of particles from the 25 spores (Table S1). d) Scatter plot when sorting only the area R1 from 25 crushed spores (Table S1).

DNA was extracted from each of the tubes using the Genomic DNA from Plant kit (Macherey-Nagel, Germany) in order to obtain clean DNA that could be tested for fungal presence. The protocol was adapted to the characteristics of the samples having low amounts of material and no cell biological material to homogenize, and encompassed the following: step 1, 125 µl of buffer MC1 and 2.5 µl of RNAse A; step

2 was eliminated, but a short centrifuge of 30 s at 5800 xg was added to bring down the evaporates; step 3, 7.5 µl magnetic beads and 100 µl MC2; step 4, 140 µl MC3; step 5, 140 µl MC4; step 6, 125 µl of 80% ethanol; step 7, 125 µl MC5; step 8, 30 µl MC6. Nanodrop was used to determine the amount of DNA in the samples after the DNA extraction (Table S1).

Table S1. Number of particles collected for each region and assessment of the DNA amount using nanodrop after extracting DNA from the samples.

Sample		Number of particles	Nanodrop (ng/µl DNA)
15 spores	R4	10795	21.5
	R1	778	22.7
	R2 (- R4)	4846	28.1
	All regions	-	20.8
25 spores	R4	16000	26.0
	R1	1300	28.2
	R2 (- R4)	8000	22.5
	R1	622	26.4
	All regions	-	25.6

Finally, to resolve which of the regions of the scatter plot the represent fungal nuclei, PCR was performed using fungal specific primers ITS1F/ITS4 [63] for amplification of the rDNA ITS region. Reaction mixture contained 5x Buffer HF (Qiagen, Sweden), 2 mM MgCl2, 0.2 mM deoxynucleoside triphosphates (dNTPs), 0.4 mM DMSO, a 0.25 µM concentration of each primer, and 1 U Phusion DNA polymerase (Qiagen, Sweden). The PCR protocol included an initial denaturing step of 30 s at 98°C, followed by 35 cycles of 30 s at 98°C, 30 s at 57.5°C, and 30 s at 72°C, before a final elongation step of 10 min at 72°C. The reaction was performed with a 2720 Thermocycler of Applied Biosystems (Fishers Scientific, Sweden). Amplification products were separated by gel electrophoresis (1.5 % agarose) at 70V for 80 min and visualized using the imaging system Molecular Imager Gel Doc XR+ (Bio-Rad Laboratories, USA) and the software Image Lab v 4.1 build 16 (Bio-Rad Laboratories, USA). Based on the gel visualization we concluded that fungal nuclei were most frequently appearing in the region R1 of the scatter plot, as these samples generally showed stronger amplification of the fungal barcode region (Figure S3).

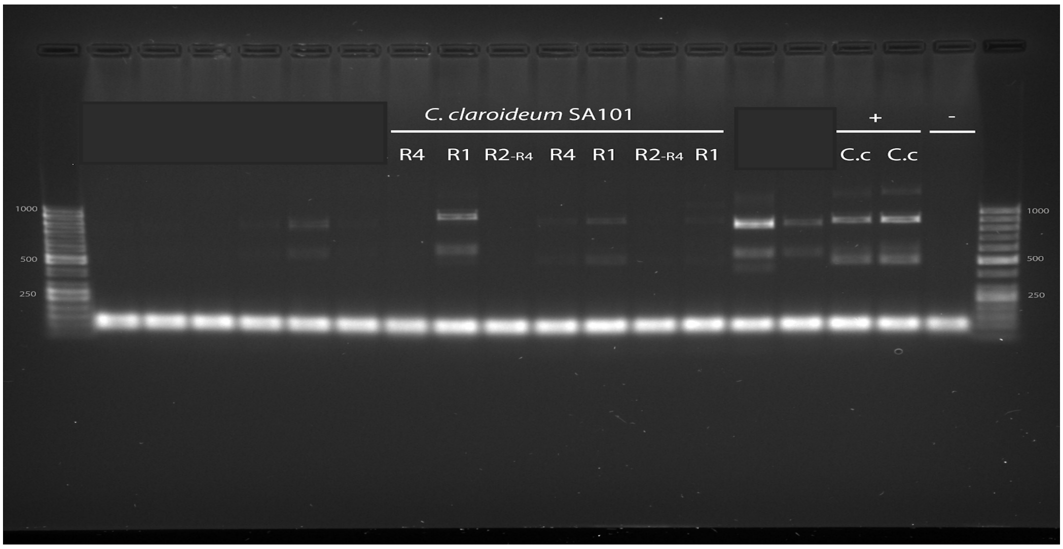

Figure S3. Agarose gel showing the PCR products for particles collected from the scatter plot regions R1, R2-R4, R4 as well as a pool of all regions as positive control sorted from *C. claroideum /C. luteum* (SA101). Gel electrophoresis was done using a 1.5% agarose gel, run for 80 min at 70 V. Thermo Scientific GeneRuler 50 bp DNA Ladder was used. For easier visualization, samples that belong to other samples have been covered, no further modification was done, for original image see Figure S7.

<u>Nuclei extraction and sorting</u>

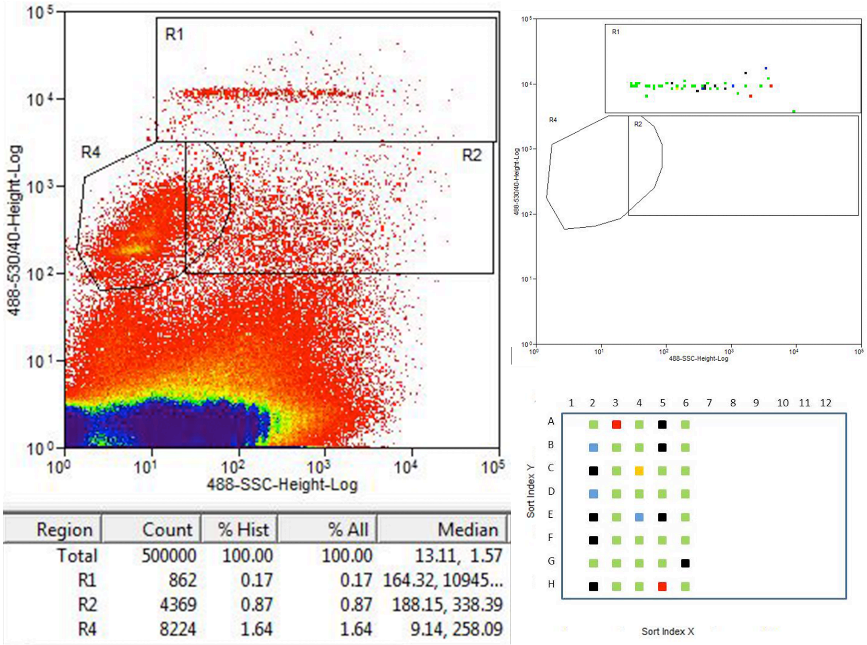

Region	Count	% Hist	% All	Median
Total	500000	100.00	100.00	13.11, 1.57
R1	862	0.17	0.17	164.32, 10945...
R2	4369	0.87	0.87	188.15, 338.39
R4	8224	1.64	1.64	9.14, 258.09

Figure S4. To the left, scatter plot of particles from one single crushed spore from which the R1 region was sorted into wells. Two right panels, the particles that were sorted and were further tested for fungi/bacteria. Every particle can be traced back from the plate to the exact sorting event. Colors represent the obtained results observed in Figure S5: fungal positives/bacteria negatives (green), fungal positives/bacteria positives (blue), fungal negatives/bacteria positives (red), no amplification (black).

Table S2. 96 well plate layout for sorting. In blue, empty wells as negative controls; in light orange, single particles; in dark orange, 5 particles for positive control. Half plates were filled to reduce risk and cost in downstream handling.

	1	2	3	4	5	6	7	8	9	10	11	12
A		1	1	1	1	1						
B		1	1	1	1	1						
C		1	1	1	1	1						
D		1	1	1	1	5						
E		1	1	1	1	1						
F		1	1	1	1	1						
G		1	1	1	1	1						
H		1	1	1	1	5						

<u>Selecting single amplified nuclei for sequencing</u>

From the sorted particles nuclei were selected for sequencing when the fungal barcode region amplified but not the bacterial region (Figure S5, Table S3). The DNA concentration of the 24 selected nuclei was estimated using Qubit (Invitrogen, Austria) (Table S4).

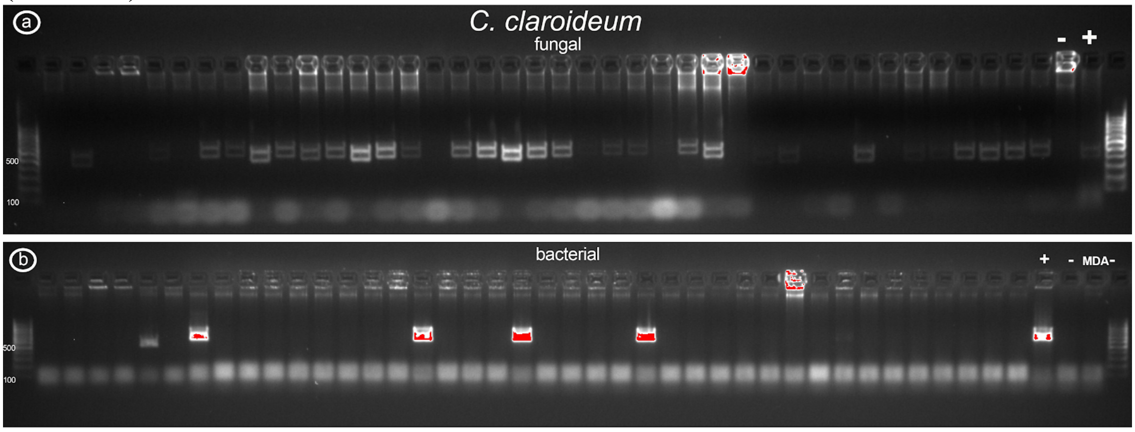

Figure S5. PCR amplification of sorted and MDA single nuclei for a) Fungi (positive control *Agaricus bisporus,* negative control ddH_2O) and b) Bacterial (positive control *Legionella,* negative control ddH_2O). Gel electrophoresis (2% agarose gel) run for 35 min at 110 V and 70 V respectively with Thermo Scientific GeneRuler 100 bp DNA Ladder. Gels images have been cropped to show only the samples of this study, no further modification was done, original images (Figure S8-S9.

Table S3. Total number (% of total) of sorted and amplified nuclei samples within the four categories Fungi +, Bacteria +, Fungi and Bacteria + and Failed/Empty, based on PCR scoring described above. Total number sorted and tested in the last column.

Species	Fungi +	Bacteria +	Fungi & Bacteria +	Failed/Empty	Total
C. claroideum/C. luteum (SA101)	27 (67.5%)	2 (5%)	3 (7.5%)	8 (20%)	40

Table S4. DNA concentrations of whole genome amplified DNA from 24 sorted nuclei of *C. claroideum/C. luteum* (SA101) that were selected for sequencing.

Sample	Well code	DNA concentration (ng/µl)
1	A2	24.5
2	B2	26.0
3	D2	22.5
4	C3	23.3
5	D3	23.0
6	F3	23.2
7	G3	23.8
8	H3	24.9
9	A4	22.2
10	B4	23.8
11	C4	23.7
12	D4	26.2
13	E4	22.6
14	F4	26.5
15	G4	21.7
16	H4	23.7
17	C5	22.6
18	D5	26.4
19	G5	23.2
20	A6	27.2
21	B6	21.5
22	D6	23.6
23	E6	21.4
24	F6	23.8

Raw read quality control

In the OSF repository for the project[44] all quality control results for the raw reads are deposited, as well as the qualitative and quantitative assessments of the assemblies.

The standard of Minimum Information about a Single Amplified Genome (MISAG) developed by the Genomic Standards Consortium (GSC)[64] was used as a guideline to assess the quality of the assemblies (Table S5). The highest quality obtained in this study is a high-quality draft, considering that the final assembly still contains gaps spanning repetitive regions. Exploring the effect of short contigs in assembly workflow 1 and found that contigs <2000bp tend to inflate genome size due to duplications (Figure S6, Table S6).

Table S5. Single nuclei assembly statistics for the assembly workflow 1 (raw reads assembled with MaSuRCA[34]) and 2 (normalized reads assembled with SPADES[35]). Assembly size (Mb), number of contigs, N50, size of largest contig and % of raw reads covered by the individual assembly is presented for each nucleus (1-24).

	Assembly workflow 1 (raw reads + MaSuRCA)					Assembly workflow 2 (normalized reads + SPADES)				
nucleus	Size (Mb)	# Contigs	N50	Largest contig (Kb)	% or raw reads mapped	Size (Mb)	# Contigs	N50	Largest contig (Kb)	% or raw reads mapped
1	35.44	16406	3231	36.3	53.44	27.9	7592	8568	58.9	95.52
2	31.29	15557	2959	33.1	54.11	24.90	6609	9212	199.9	96.81
3	25.03	16526	1672	13.2	50.77	20.14	7764	5294	61.2	95.99
4	31.10	14480	3149	25.6	52.36	22.98	6175	8841	62.4	95.66
5	39.00	17793	3351	32.9	55.70	27.72	7429	9343	78.0	95.29
6	36.19	16212	3510	46.6	51.94	27.38	7453	9824	63.8	95.87
7	56.06	26206	3199	31.9	52.88	41.98	9863	10068	95.0	95.82
8	51.87	23590	3237	38.9	51.08	39.23	9291	10447	60.6	95.83
9	42.24	19220	3284	30.1	52.42	30.72	8355	9500	71.7	95.86
10	51.87	23590	3237	38.9	61.59	16.74	5150	7065	55.5	96.11
11	14.47	7425	2705	22.5	48.11	11.78	3500	7214	41.2	96.31
12	45.21	21122	3120	42.3	53.31	33.37	8415	9772	74.8	95.74
13	56.85	24537	3785	38.2	60.21	40.47	10218	11172	147.0	96.47
14	18.19	10279	2237	22.6	55.10	14.80	4644	6635	41.9	96.29
15	22.47	14432	1804	14.2	60.83	18.47	10816	3085	38.4	94.15

16	38.52	20163	2471	25.1	60.28	27.71	8949	6448	60.8	94.67
17	40.72	17740	3683	40.5	58.43	29.54	8651	9334	67.5	95.64
18	57.06	23828	3901	41.7	64.69	39.50	9940	10143	76.4	95.29
19	35.45	16004	3472	43.7	54.52	25.99	7608	9149	66.2	95.59
20	43.45	18217	3945	39.6	59.13	31.28	8841	9276	63.8	95.68
21	28.37	13758	2951	23.8	52.03	21.00	5886	8353	81.1	95.62
22	69.45	30847	3437	41.6	57.94	50.24	11265	11295	71.6	95.36
23	36.76	17998	2806	29.7	46.29	28.75	7364	9287	81.5	96.20
24	49.34	22402	3254	30.2	64.52	34.97	9870	8227	66.4	95.41

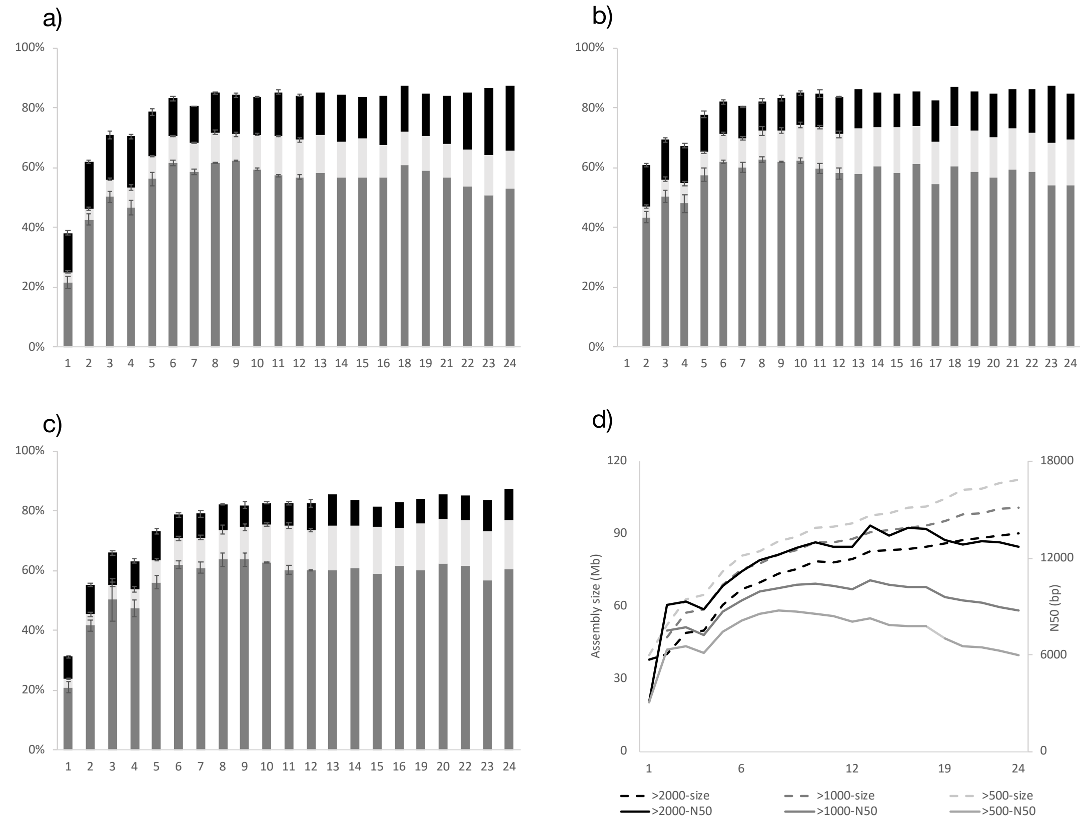

Figure S6. Summary statistics for different number of assembled nuclei (1-24) using assembly workflows 1 based on raw reads of individual nuclei assembled using Masurca, consensus assembly using Lingon. BUSCO estimates of completeness for a) workflow 1 contigs >500bp, b) workflow 1 contigs >1000bp, and c) workflow 1 contigs >2000bp. The later is the best option of the three, presented as method 1 in the main text. Percentage of single copy core genes detected as single copy (S: grey), duplicated (D: light grey) or fragmented (F: black). Average of 3-6 replicate assemblies up to 12 nuclei with error bars indicating SEM. In d) assembly size (dashed lines) and N50 (solid lines) for the there methods 1 >2000 bp (black), >10000 bp (grey) and >500 bp (light grey).

Table S6. Presence of the single copy genes EF1 and RPB1 in the generated assemblies. Present as single copy in both assembly methods 1 and 3, but not in assembly method 2. Contigs and regions where the gene is found are shown on the table.

	EF1		RPB1	
Assembly	Contig	Region	Contig	Region
1	contig006909	1349-2165	contig004936	7862-10400
1n	contig003719	30811-31627	contig003448	7862-10400
2	contig010744	1152-1968,<br>14549-15365	contig003808<br>contig001875<br>contig011350	7613-10151<br>1-1098<br>1-638
2n	scaffold_376	49952-50763,<br>63349-64165	scaffold_1261<br>scaffold_339<br>scaffold_1645	9366-11904<br>36857-37954<br>3258-3895
3	contig000025	24541-25357	contig000552	15574-18112
3n	scaffold_129	24541-25357	scaffold_994	15574-18112

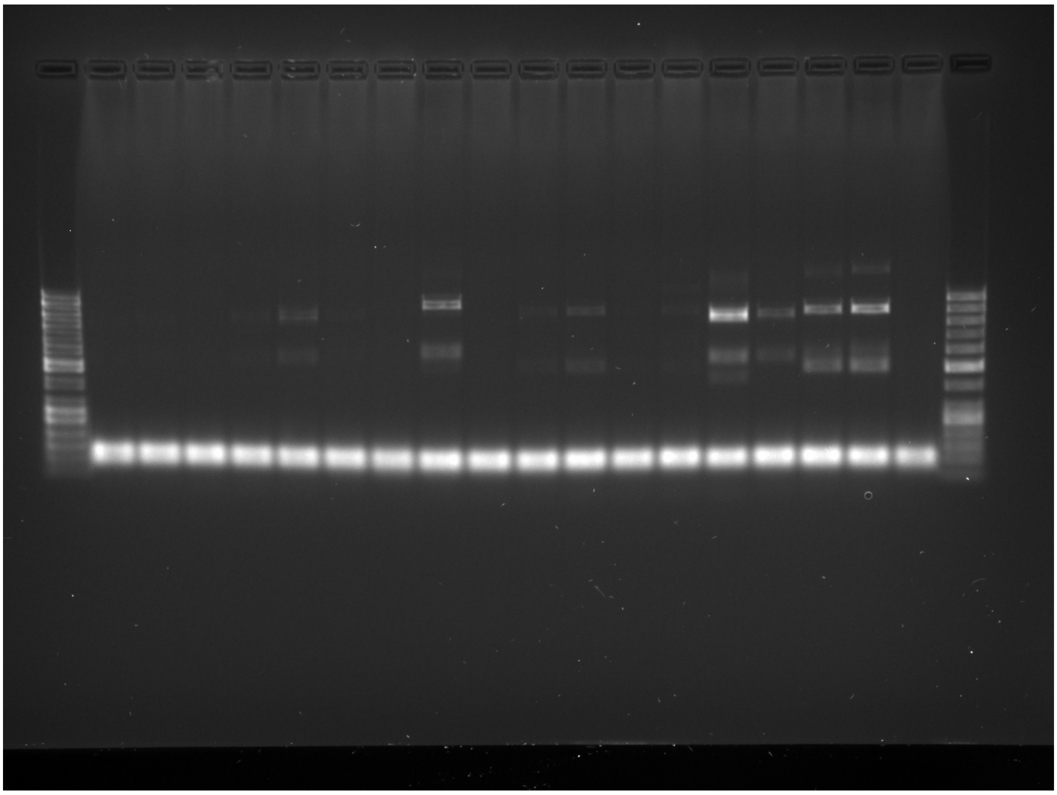

Figure S7. Original gel image corresponding to figure S3. Image taken using the imaging system Molecular Imager Gel Doc XR+ (Bio-Rad Laboratories, USA) and the software Image Lab v 4.1 build 16 (Bio-Rad Laboratories, USA).

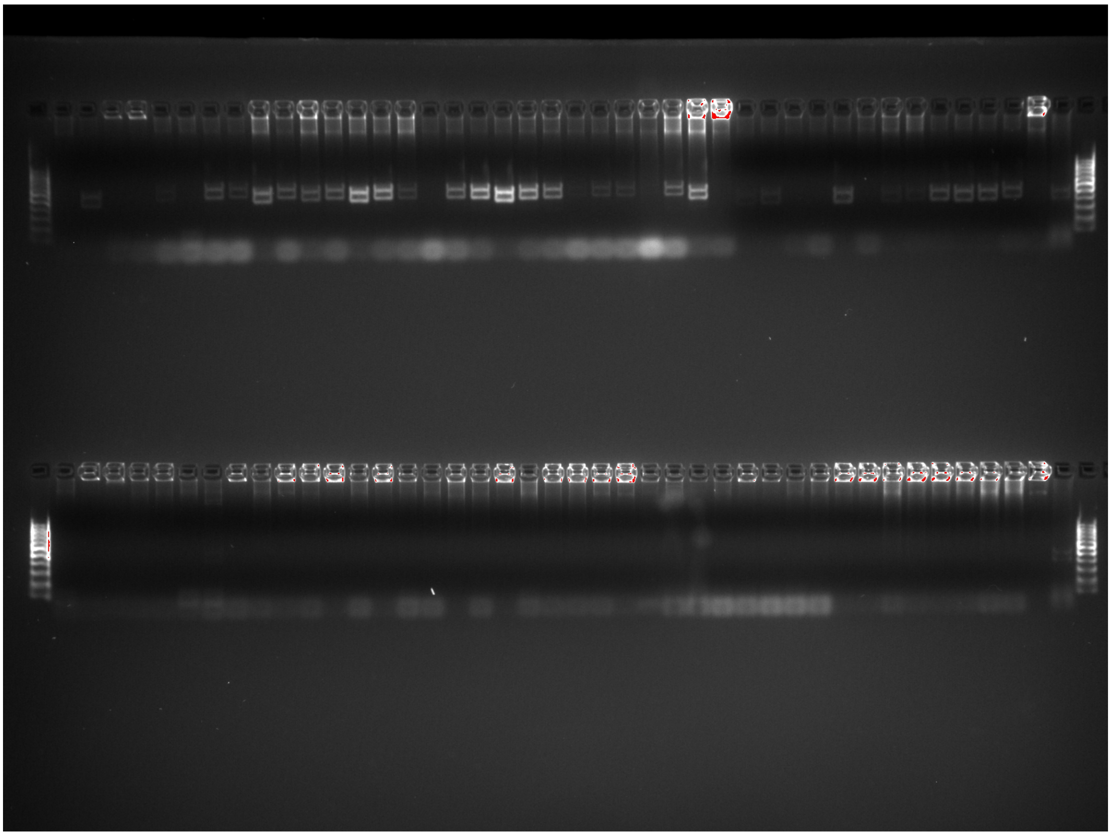

Figure S8. Original gel image corresponding to the upper part of figure S5. Image taken using the imaging system Molecular Imager Gel Doc XR+ (Bio-Rad Laboratories, USA) and the software Image Lab v 4.1 build 16 (Bio-Rad Laboratories, USA).

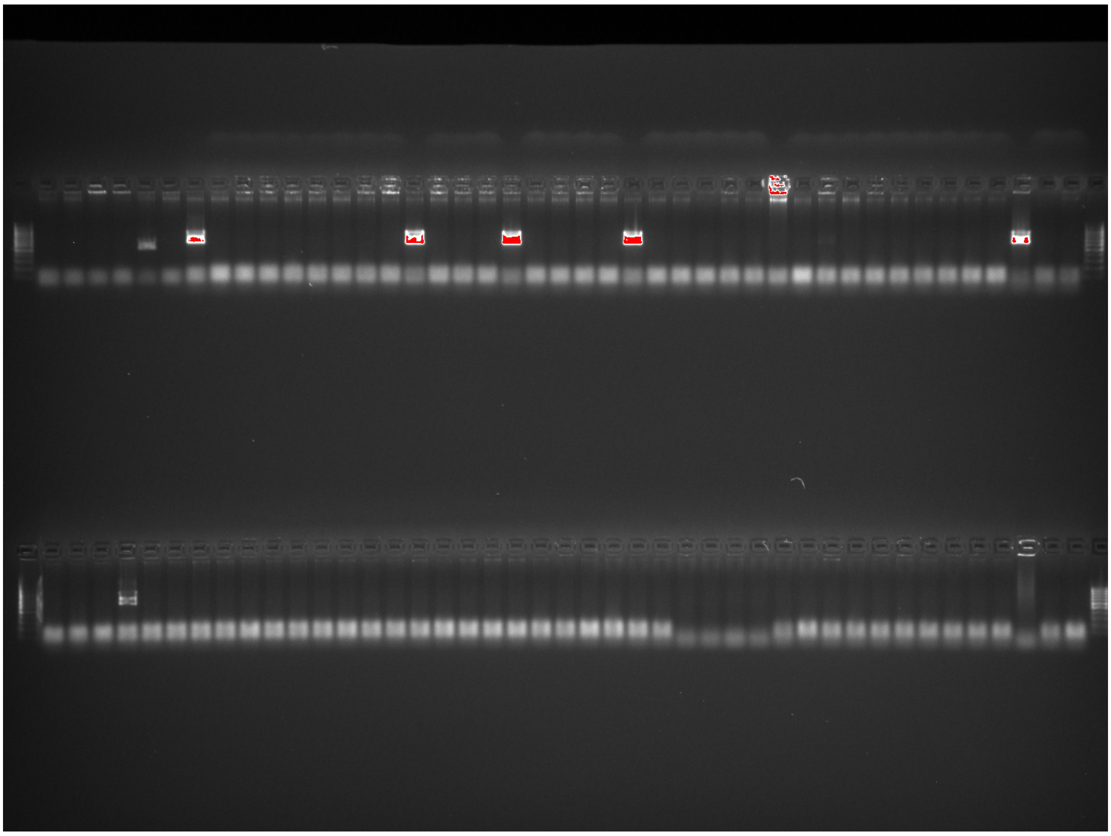

Figure S9. Original gel image corresponding to the lower part of figure S5. Image taken using the imaging system Molecular Imager Gel Doc XR+ (Bio-Rad Laboratories, USA) and the software Image Lab v 4.1 build 16 (Bio-Rad Laboratories, USA).
